# Designing a multi-arm, multi-stage platform trial for venous leg ulceration – Venous leg ulcers: management and eradication, the VEIN platform study. Designing a platform/adaptive trial for venous leg ulcers (VLU) – The VEIN platform

**DOI:** 10.3310/nihropenres.13847.1

**Published:** 2025-04-14

**Authors:** Sarah Onida, Alun Huw Davies, VEIN Platform consortium, Sarah Onida, Alun H Davies, Athanasios Saratzis, Dan Carradice, Francine Heatley, Laura Burgess, Layla Bolton-Saghdaoui, Rachel Phillips, Shaun Barber, Nicholas Johnson, Thomas M Withers M Withers, Jo Dumville Dumville, David Epstein Epstein, Justin Waring Waring, Colin J Greaves J Greaves, Steven K Rogers, Adarsh Babber, Manjit Gohel, Ian Chetter, Leanne Atkin, Salvatore Di Puma, Narayan Karunanithy, Stephen Black, Bernard Ho, Nina Al-saadi, John Norrie, Andrew Stoddart, Babak Choodari-Oskooei, Max Parmar, Marcus Jepson, Kavita Vedhara, Barnaby Carter, Natalia Klimowska-Nassar, Ed Waddingham, Gordon W Moran, Colin Crooks, Azeem Majeed, David Wingfield, Matthew R Sydes, Adam Gwozdz, Vivien Gibson, Andy Steptowe

**Affiliations:** 1 Imperial College London Department of Surgery and Cancer, London, England, UK

**Keywords:** Venous leg ulceration (VLU), healed ulceration, venous disease, randomized controlled trial, platform trial

## Abstract

**Background:**

Venous leg ulceration (VLU) is the most severe form of venous disease and an important burden to patients and society. Many treatments for VLU exist, including wound therapies, medications, and surgical interventions. However, high-level randomized trial data supporting leg ulcer treatments are lacking, limiting their adoption in clinical practice. We developed a platform trial assessing multiple interventions for VLU comprising multiple multi-arm multi-stage trials.

**Methods:**

Scoping reviews, surveys, meetings, and focus groups were conducted over five work streams to inform the development of the proposed platform trial. We involved international experts in venous disease, patients with a lived experience of VLU, healthcare professionals with an interest in VLU care, methodologists, industry partners, and other key stakeholders to help inform priority research areas and methodology, and finalize the proposed trial design.

**Results:**

Based on this feedback, the proposed multi-arm multi-stage (MAMS) platform trial will be delivered across three patient domains: patients with active ulceration present for less than six months (Domain 1), patients with active ulceration present for more than six months (Domain 2), and patients with healed ulceration (Domain 3). Interventions included wound care, medication, intervention for superficial venous reflux, and surveillance strategies delivered across community, primary, and secondary care.

**Conclusions:**

Our MAMS platform trial development highlighted many challenges and opportunities in methodology development and the potential delivery of such a study. The work performed in our work streams will help inform future research in this field.

## Plain Language Summary

**Background:**

Veins are responsible for draining blood into the circulatory system and transporting it back to the heart. When they do not work in the lower limbs, the blood pools in the legs and leads to high pressure and skin damage. In some cases, wounds, known as venous ulcers, can develop. These are more common in the elderly and can be extremely difficult to heal. Even when they heal, there is still a high risk of recurrence. Patients with these ulcers suffer from pain, embarrassment, and report a negative impact on their lives. Venous ulcers are also very expensive, with the cost of their care more than double the combined cost of treating lung, bowel, breast, and prostate cancers. The number of ulcers is increasing, and there is an urgent need to develop methods to treat them more efficiently.

Many treatments are available for these ulcers. However, good research supports the use of tight bandages or surgery to treat veins that do not work. There is little to no research supporting the use of all other possible treatments. Because of this, we do not use them routinely to treat ulcers, and we do not know whether they are effective. Many of these treatments are simple and can easily be used to treat patients outside the hospital. They could potentially benefit many patients if they are shown to work. In this study, we tested these different treatments.

**Aim:**

During this research, we aimed to answer the following question: *What is the ideal study to test multiple different treatments for venous leg ulcers?* To answer this question, we developed a National Institute for Health Research (NIHR) grant application for the proposed study.

**Research plan:**

We used different research approaches to answer this question across five work streams (WS). This included searching for all previously published treatments for venous ulcers to ensure that we had a complete evidence base (WS1), involving clinicians, patients, companies, healthcare professionals, and statisticians to have the best experts contributing to the study (WS2), identifying the main research questions and key priorities for the research (WS3), developing and finalizing the research proposal (WS4), and submitting an application for funding the proposed study (WS5). This was performed with surveys, focus groups, and meetings in various parts of the country to obtain as many opinions as possible on the research.

**Knowledge mobilisation:**

We planned to send monthly study bulletins to all the collaborators and patients involved in the study development, updating them on study progress. We planned to publish our study findings as soon as they became available. We planned to communicate study updates through national bodies, national and international meetings, and patient groups. We submitted an application for funding to the NIHR, which progressed to stage 2, but was ultimately not funded. The information obtained during this process will inform future research on venous leg ulcers.

## Introduction

Venous leg ulceration (VLU) is the most severe manifestation of chronic venous disease and the most common cause of lower limb ulceration. The CEAP classification ^1^ is an international staging system for venous disease that describes active ulcers as class C6 and healed ulcers as class C5. The prevalence of VLU is increasing with the aging population and presents a significant burden on patients and healthcare services. Those affected experience chronic pain, sleeplessness, and social embarrassment, leading to a major negative impact on their quality of life. Although VLU affects approximately 1% of the general population, it disproportionately affects the elderly, with over 4% of those aged > 65 years affected ^2^. This condition is very expensive and is responsible for an expenditure of over 1% of the annual healthcare budget, costing over £3.2 billion per annum ^3, 4^. This is more than double the combined annual cost of managing breast, colorectal, prostate, and lung cancer ^5^.

A wide variety of treatments exists for VLU, including medical therapy, exercise, and interventional treatment. However, high-level evidence exists for only a limited number of management strategies, resulting in limited evidence-based treatment options. Currently, VLU treatment focusses on the application of compression therapy and intervention to ablate refluxing superficial veins ^6, 7^. This aims to reduce ambulatory venous pressure in the lower limb, which is the cause of skin damage and ulceration. Compression and superficial endovenous ablation are supported by high-level randomized controlled trials ^8, 9^. Specifically, the EVRA trial found that early ablation of superficial refluxing veins significantly reduced healing time and increased ulcer-free time compared with delayed ablation ^9^. The ESCHAR trial found that surgical intervention significantly reduced twelve-month ulcer recurrence rates, although there was no difference in ulcer healing compared with compression alone ^10^.

Despite these interventions being embedded in both National Institute for Health and Care Excellence (NICE) and international guidelines ^6, 7^, non-healing and recurrence rates are still unsatisfactory. Non-healing rates are reported to be 20–40%, with recurrence rates as high as 40% at three years in randomized controlled trials ^11, 12^. Real-world data suggests that these rates are significantly higher ^13^. This is a major concern in the population of older, vulnerable people with multiple long-term conditions.

This was recognized nationally in the James Lind Alliance vascular priority setting partnership ^14^ and by the All-Party Parliamentary Group on Venous Diseases ^15^, which highlighted VLU research as an urgent priority for the NHS.

There is an urgent need to develop high-level evidence for various VLU treatments to improve outcomes in the VLU population. The aim of the VEIN accelerator award was not only to develop an efficient and optimized trial design that allowed the testing of several existing VLU treatments, but also to introduce and assess new therapies in the context of a platform trial.

## Methods

### Patient and Public Involvement

All patients were involved in the development of this study. We conducted online and person-focus groups across the country to involve as many patients as possible. The main research questions, treatments, outcomes, and follow-up methods were developed on the basis of patient feedback. Two of the final study co-applicants were patients with a lived experience of venous ulcers who reviewed the final application to ensure that this was relevant to patients. The final study proposal was developed with methods that allow for easy input and feedback by patients with venous ulcers throughout the planned research.

**Overarching aim**: To identify the optimal design for research delivery for a large-scale, randomized, multi-arm, multi-stage platform trial assessing the clinical and cost-effectiveness of multiple interventions for venous leg ulcer healing and recurrence.

This research was a grant development exercise and a service evaluation exercise. No ethical approval was required for this research.

The research was conducted in five work streams (WS) with individual aims, as described below:

### Work stream 1: Evidence synthesis and identification of research priority areas

A scoping review of the literature was planned to collect evidence on all published treatments for VLU. In addition, specific systematic reviews were planned to provide evidence for individual treatment modalities of particular interest. The aim of this WP was to identify potential treatments for inclusion in a platform trial.

### Work stream 2: establishment of the platform development group

The aim of this WS was to identify key individuals to help inform the development of the platform. This was planned across tiers of involvement.

1. The executive group informed the day-to-day development of the platform via weekly meetings, including:

- Joint lead applicants
- Lead statistician
- Project manager
- Lay patient co-applicants

2. Core groups directly informed the development of the platform trial through regular meetings (monthly)

- Core patient group: Patients, caregivers, and family members with lived experience of VLU
- Core clinical group – clinicians with expertise in VLU management
- Core stakeholder group – representatives from industry, statisticians, health economists among others

3. Specialist advisory groups (SPAGs) indirectly periodically informed platform development via surveys and questionnaires.

- Patient representatives – to obtain additional, diverse patient and carer feedback
- Clinical representatives – to obtain diverse input from clinicians internationally
- Industry partners – to ensure that the latest developments in medical technology were brought to the attention of the platform.
- Statistical expertise – to support complex adaptive statistical analyses required for the platform
- Health economic expertise – to examine the cost effectiveness of the proposed interventions
- Qualitative expertise – to support qualitative data analysis during the platform development
- Trainee collaborative – to ensure inclusion and involvement of trainees in the research
- Data science expertise to maximize the inclusion of evidence-based health informatics in the platform

### Work stream 3: establishment of key performance indicators (KPIs) and characteristics of the platform trial

The aim of WS3 was to identify the key characteristics of future platform trials, including

- Study design
- Patient centric methodology
- Governance structure
- Health economic and statistical methodology
- Data analytics
- Dissemination plans
- Equality and diversity

### Work stream 4: consensus one the optimal design, methodology and delivery of the research

The aim of WS4 was to obtain a consensus on the final design and methodology based on the results of the previous work packages:

- The finalised patient, intervention, comparison, outcome (PICOT) design of the proposed platform
- The finalised KPIs and milestones for the platform trial delivery
- Key plan for the proposed integration with existing routine healthcare datasets, such as clinical practice research datalink (CPRD), hospital episode statistics (HES), and office for national statistics (ONS)
- Establishment of how early success and futility would be identified to add and remove trial arms
- Establishment of how interactions between treatments would be accounted for
- Identify ways to disseminate the research in a rapid manner

### Work stream 5: finalising the funding application

The aim of WS5 was to deliver a finalized funding application for the proposed platform trial to the National Institute for Health and Care Research (NIHR) in November 2023.

### Design and theoretical framework

The study employed a mixed methods approach, including evidence synthesis (WS1), interviews, questionnaires, focus groups (WS 2–3), statistical simulation to identify the most efficient design and sample size calculation, and model scoping and simulation for cost-effectiveness (WS4). We modelled our platform trial development according to published practical guidelines ^16^ for platform studies.

The initial work streams were proposed following meetings with lay co-applicants with a lived experience of VLU. The five WS were further developed with weekly meetings by the executive group with regular input from other members of the platform trial governance (core groups, SPAGs). We ensured widespread representation with patients and caregivers from across the UK and international experts involved in the development of the platform. Methodology expertise was sourced from three clinical trial units (CTU: Imperial, Leicester, Edinburgh), with the support of a fourth unit with expertise in designing platform trials (medical research council [MRC] CTU at UCL) and a strong track record of research delivery. In addition, partners from the Vascular Society of Great Britain and Ireland, Royal College of Surgeons of England, Venous Forum of the Royal Society of Medicine, European College of Phlebology, Vascular Research UK, and Vascular and Endovascular Research Network supported this research.

### Work stream 1: Evidence synthesis and identification of research priority areas

The first WS started upon the confirmation of funding. A scoping review was performed using the following databases: EMBASE, MEDLINE, Cochrane database, and clinicaltrials.gov using Medical Subject Headings (MeSH) terms pertaining to VLU healing and recurrence, permitting the identification of:

- Complex trials in patients with VLU (ongoing or complete)
- Interventions in VLU patient care to inform possible trial arms (including pharmacological, topical, and interventional).
- Comparators in VLU trials to inform the definition of the platform control group
- Outcomes reported in VLU trials to inform possible outcomes in the platform

Following the scoping review, further in-depth systematic reviews and meta-analyses were conducted on interventions that were felt to be possible candidates for the trial arms to assess the existing evidence base in greater detail.

NHS library resources were used for all reviews. Team members with expertise in scoping and systematic reviews performed the searches, title, and abstract screening, including searches of reference lists. The analysis was performed, and the results were disseminated to our lay co-applicants.

### Work stream 2: Establishment of the platform development group

Concurrent with the evidence synthesis, a platform development group was established. The co-applicants were keen to obtain a broad range of inputs during platform development, while also being aware of the need for robust governance to ensure input could be efficiently and meaningfully incorporated into trial development.

We established three tiers of involvement in the platform development:

1. The executive group included core co-applicants, such as joint lead applicants, key statisticians from the CTUs, project managers, and two named patient co-applicants with lived experience of VLU. This group convened weekly to ensure that the research progressed.
2. Core groups: These groups included diverse patients with a lived experience of VLU, key experts in the field of VLU, and additional stakeholders who met on a monthly basis and provided feedback on the PICOT framework for the proposed trial.
  a. Core patient group: We selected a diverse patient group recruited among volunteer patients in the outpatient clinics of collaborating institutions. This included 14 patients with lived experience of VLU selected to ensure diversity in terms of sex, ethnicity, geographical location, and socioeconomic background.
  b. Core clinical group: We established a group of international clinicians with expertise in VLU research using our existing research network. The experts had backgrounds in nursing, vascular surgery, dermatology, interventional radiology, physiotherapy, pharmacy, and general practice.
  c. Core stakeholder group: This group was established via existing international links and included industry partners, statisticians, health economists, and data science experts.
3. Specialist advisory groups (SPAGs) – these groups provided additional input into the platform by advising the core groups on platform development via surveys, questionnaires, and focused interviews, where appropriate. They included
  a. Patient representatives – We recruited a large pool of patients who were willing to participate in questionnaires and focus groups, and provided anonymized feedback via a web interface.
  b. Clinical representatives: We included national and international society representation championing VLU care and research, including the American Venous Forum, American Vein and Lymphatic Society, Royal Society of Medicine, European College of Phlebology, International Union of Phlebology, European Venous Forum, National Wound Care Strategy Programme, and Legs Matter.
  c. Industry partners: We invited company representatives to help support the introduction of medical devices and/or pharmacological agents in the trial arms.
  d. Statistical expertise – members of the three CTUs.
  e. Health Economics Expert – Health Economists from the University of Granada and University of Edinburgh.
  f. Qualitative expertise – experts in qualitative methodology, research, and behavioral health were included in all meetings to help identify themes and categories and ensure that the maximum amount of data was distilled from group encounters.
  g. Trainee collaboratives: We are keen to promote trainee involvement in VLU research. Therefore, we included the Vascular and Endovascular Research Network (VERN), a well-established trainee-led collaborative that has successfully delivered international studies.
  h. Data science – Recognizing the value of routine healthcare data, we wanted to ensure integration of electronic healthcare records in the platform, particularly for longer-term data points. We established a collaboration between the British Heart Foundation (BHF) Consortium and the Big Data Analytical Unit (BDAU) at the Imperial College London.

### Work stream 3: definition of Key Performance Indicators (KPIs) and platform trial characteristics

We performed online focus groups and interviews with the core patient group to inform them of the key priorities from the patient’s perspective. The core patient group, comprising volunteers identified in the participating institutions, participated in two online focus groups, where priorities for the research and possible trial arms were discussed. Although we aimed to maximize diversity in the group, we found that individuals who volunteered for online focus groups were highly educated and well-IT-versed. We developed concerns that the group was not diverse enough from a socioeconomic perspective. Previous research has shown that VLUs are often found in patients experiencing socioeconomic deprivation. Therefore, we decided to develop additional face-to-face patient and public involvement (PPI) events in London and Wakefield to ensure socioeconomic diversity. Similar to the core patient group, these participants were recruited in the outpatient clinic and varicose vein lists of collaborating institutions, but were provided with the option to participate in in-person meetings. These were attended by 20 individuals, improving the diversity of our patient group.

The core clinical groups routinely met on a bi-monthly basis to discuss the results of WS 1 and develop the key platform characteristics with periodic input from the SPAGs via online questionnaires. The following areas were e discussed:

- Study design
- Patient centric methodology
- Governance
- Health economic and statistical methodology
- Data analytics
- Dissemination plans
- Equality and diversity

### Work stream 4: consensus on the optimal design, methodology and delivery of the research

In WS4, we reached a consensus on the research design. This was performed via online meetings between the executive and core groups with input from SPAGs. During this WS, we finalized the design of the research, including the PICOT framework (Population, Intervention, Comparator, Outcome, Timeframe), establishing the KPIs to assess research delivery and milestones, consensus on how patients are identified and approached efficiently based on unique regional and national characteristics, identification of success and failure criteria for each intervention relating to safety, considerations regarding recruitment challenges, clinical and cost effectiveness, consensus on enablers and barriers for research delivery within the expected timelines, and budgets creating a vehicle for efficient and timely dissemination throughout trial delivery.

From a methodological perspective, we established analysis methods for key outcomes using a simulation-based, frequentist approach to investigate and optimize trial design, considering adaptive elements such as timing and number of interim assessments and corresponding stopping rules, discussing how national registries or cohort studies could be used to streamline trial delivery, and integration of electronic healthcare records for long-term outcome assessment.

As part of this work package, we also finalized patient-facing interfaces for platform trial information for the public. This included the development of a dedicated VEIN website with information on the platform methodology and platform arms, as well as a dedicated infographic to support patient and public engagement, promotion, and dissemination of the research.

### Work stream 5: finalising the funding application

In this WP and based on the information from WS 1–4, we prepared an application for the NIHR November 2023 deadline.

### Data collection and data analysis

Interviews, focus groups, meetings, and free-text content of questionnaire responses were analyzed by thematic analysis using a mapping approach by team members with qualitative research expertise. The statistical analysis plan was finalized during the platform development process, including the integration of large dataset analytics. Cost-effectiveness analysis was performed by two experienced health economists, including the development of a schematic for a core structure for an economic model that could be adapted for a variety of interventions in VLU, with key health states based on outcomes of interest identified in the WS.

## Results

### Work stream 1: evidence synthesis

**Figure 1.**
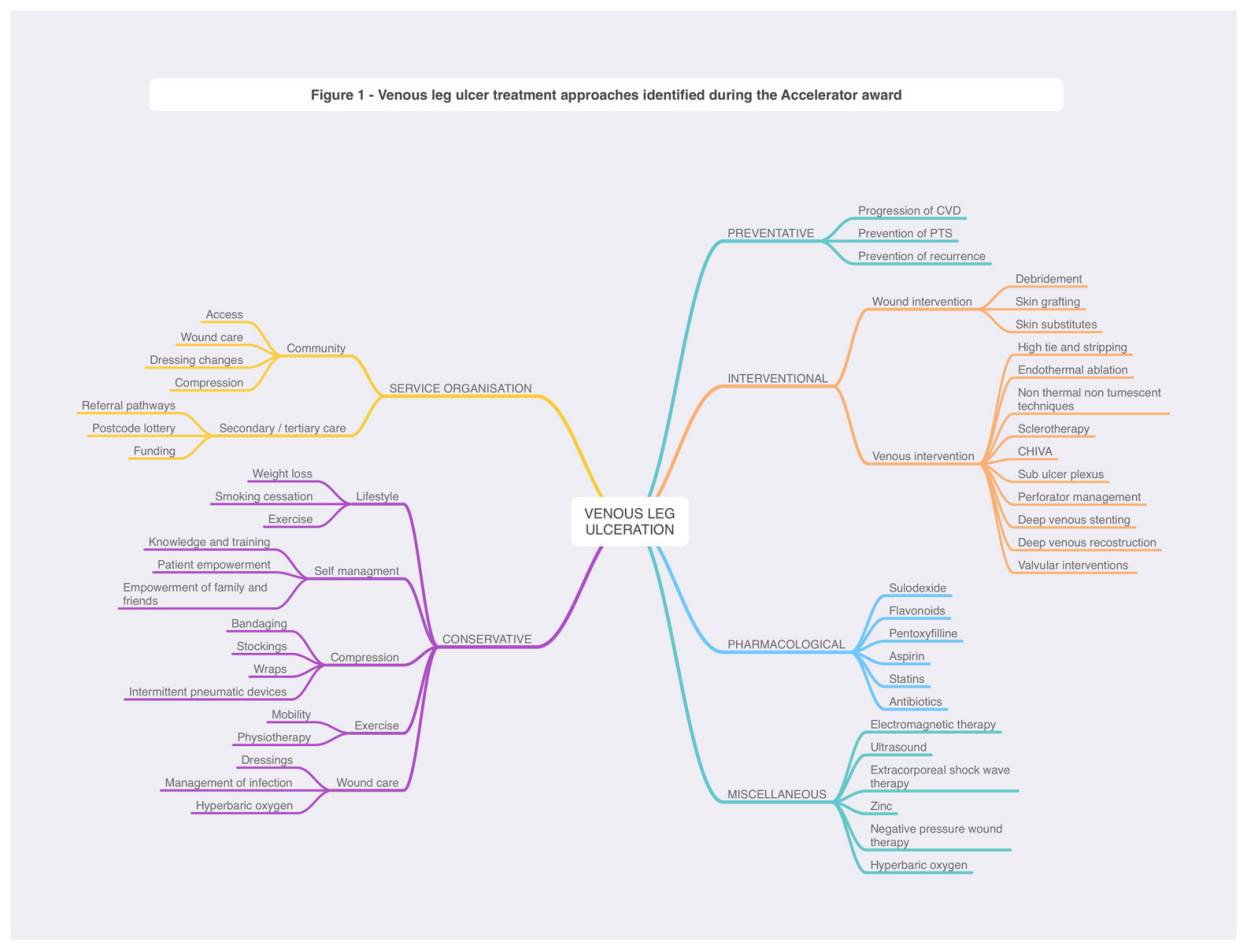
Results of scoping review.

We performed a scoping review of the literature, systematically searched MEDLINE, EMBASE, Web of Science, and CENTRAL databases, and manually searched reference lists (36,365 papers, 2,030 eligible). The scoping review identified six overall treatment approaches: conservative, pharmacological, interventional, preventive, service organization, and miscellaneous ( Figure 1). There were no published or ongoing platform/complex RCTs integrating community, primary, or secondary care. We identified a protocol paper for a study exploring the use of pentoxifylline; however, this was in combination with another oral agent and not in the context of MAMS setup.

The results were discussed in workshops and focus groups attended by platform governance members including patients and caregivers. Following consultation, we identified four priority research questions.

1. Can medications reduce the time to VLU healing and / or prevent their recurrence?
2. What is the optimal VLU wound-cleansing strategy in the community?
3. Is superficial endovenous ablation clinically and cost effective in chronic VLU?
4. Can ultrasound surveillance reduce VLU recurrence?

Further in-depth systematic reviews were performed or, if present, reviewed to obtain information on potential trial arms. Specifically, the results revealed the following:

- International guidelines highlight the potential benefits of adjunctive Pentoxifylline in VLU healing, with a number needing to treat 4.3 ^17, 18^. Our updated systematic review (ten studies, 1,025 participants) found that pentoxifylline was associated with a 62% healing rate (OR 2.56, 95% CI 1.97-3.32, p < 0.001) ^19^. We identified two studies (828 participants) on statin therapy. These results revealed that statins were associated with increased healing rates (67.6% vs. 55.6%, RR 1.23, 95% CI 1.07 – 1.41), but with significant clinical and statistical heterogeneity and paucity of data regarding recurrence. We identified statins as a potential next VEIN treatment arm. Evidence for micronized purified flavonoid fraction (MPFF) suggested that this was beneficial in VLU healing with a low risk profile, but with low quality evidence and a NNT of 7.3 to prevent one ulcer ^20, 21^. The evidence for sulodexide, cilostazol, stanazol, and aspirin use was of low quality.
- Wound-cleansing approaches are highly variable. The NICE guidelines report that the evidence for antibacterial washes is limited, of low quality, and with no data on cost effectiveness, which may be superior in terms of clinical effectiveness ^22^.
- There was no evidence on superficial venous ablation in ulcerations that were present for more than six months. The EVRA trial assessed interventions in patients with ulceration present for less than six months ^9^.
- There was no evidence on duplex ultrasound surveillance following intervention. The ESCHAR trial found that surgical intervention on refluxing veins reduced ulcer recurrence rates ^10, 11^. Duplex ultrasonography is required to identify venous incompetence recurrence, suggesting that surveillance may be beneficial in these patients.
- Additional technologies identified that may be of benefit in VLU include statins, exercise therapy, subulcer foam sclerotherapy, and assessment of long-term morbidity and mortality outcomes.

### Work stream 2: Establishment of the platform development group

We identified all key stakeholders.

### Work streams 3 and 4: Establishment of KPIs, priorities for the research and consensus on the research

**Study design:** The finalized platform trial comprised three multi-arm, multi-stage (MAMS), multicenter, prospective randomized controlled trials with an internal pilot. The trials were conducted across three domains: presence of an active ulcer for < 6 months, presence of an active ulcer for ≥ 6 months, and presence of a healed ulcer. This was believed to provide the greatest opportunity for inclusion in all patients with a history of VLU.

**Participants:** Adults aged ≥ 18 years with active ulceration or healed ulceration without peripheral arterial disease (palpable pedal pulses or ankle brachial pressure index ≥ 0.8). Domain specific inclusion criteria: Domain 1: presence of active VLU present for < 6 months Domain 2: presence of active VLU ≥ 6 months; Domain 3: healed VLU.

**Intervention:**

1. Medication – Pentoxifylline 400mg TDS
2. Antibacterial wash – Prontosan antibacterial wash
3. Endovenous truncal ablation in ulceration ≥ 6 months
4. Duplex surveillance in patients with healed ulceration

**Comparator:** standard of care (SoC) NHS

Depending on the domain, intervention and comparators ( Table 1):

**Table 1.** Intervention arms and comparators for each patient domain. SoC – standard of care; VLU – venous leg ulcer.

	Intervention Arm 1	Intervention Arm 2	Intervention Arm 3	Comparator: SoC
**Domain** **1 VLU < 6** **months**	Medication	Antibacterial wash	Combination medication + antibacterial wash	Compression; no medication; wound wash (normal saline, sterile or tap water) as per local unit protocol. For patients referred to secondary care, early endovenous ablation
**Domain** **2 VLU ≥ 6** **months**	Medication	Endovenous ablation	Combination medication + endovenous ablation	Compression; no medication; no endovenous ablation
**Domain 3** **Healed VLU**	Medication	Duplex surveillance programme	Combination medication + surveillance	Compression; no medication; no surveillance

**Outcomes:**

**Primary outcome:** Active ulceration (C6) and percentage healed (index ulcer) at 6 months. In healed ulceration (C5), the time to ipsilateral leg ulcer recurrence.

**Secondary outcome:** Quality of life (EQ5D-5L), health economic and implementation science evaluation, adverse events (AE), serious adverse events (SAE), and suspected unexpected serious adverse reactions (SUSAR) as per the MHRA guidelines for CTIMPs.

**Figure 2.**
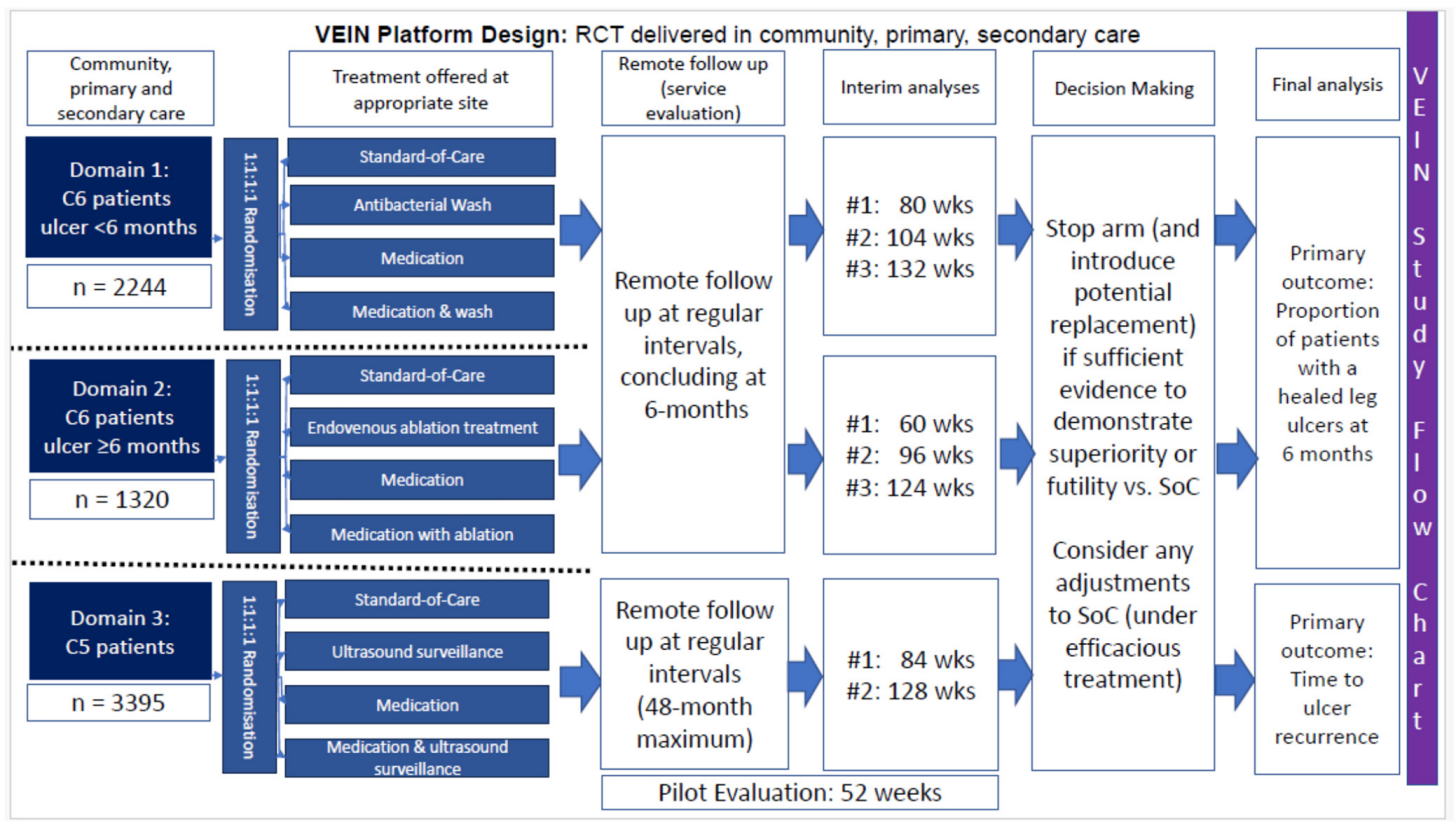
Final design of the platform trial.

**Study design:** The proposed VEIN platform trial comprised three MAMS domains running concurrently. Each domain assesses SoC against a drug-based regimen (Pentoxifylline), domain-specific intervention (Prontosan antibacterial wash, endovenous ablation, duplex surveillance), and combination therapy consisting of drug and domain-specific treatments ( Figure 2). Within the domains, participants will be randomized in a 1:1:1:1 ratio.

**Setting:** The study will be delivered in community, primary, and secondary/tertiary care, both in terms of recruitment and delivery of the trial interventions.

**Inclusion/ exclusion criteria**: Participants aged ≥18 years with a history of VLU recruited from community, primary, or secondary/tertiary care. All eligible patients will be recruited without exclusion based on their socioeconomic or ethnic backgrounds. Domain specific criteria include: Domain 1: Presence of active VLU (C6) present for < 6 months; Domain 2: Presence of active VLU (C6) present for ≥ 6 months; Domain 3: Patients with healed VLU (C5). Exclusion criteria: Patients without an active or healed ulcer, and patients with a diagnosis of peripheral arterial disease (impalpable peripheral pulses or ABPI <0.8).

**Technologies assessed:**

**Pentoxifylline:** this is a phosphodiesterase inhibitor, which reduces inflammation, blood viscosity, and platelet aggregation. At a dose of 400 mg three times a day, VLU healing rates improved by 21% when used as an adjunct to compression and by 23% with medication alone ^17^. NICE mentions pentoxifylline as a treatment adjunct for VLU, although its use is unlicensed. Existing evidence is of variable quality, and high-quality studies are required to assess the clinical and cost-effectiveness of this treatment.

**Antibacterial wash:** Prontosan® is an antibacterial preparation (Betaine and polyhexanide) available in NHS practice indicated for the prevention and removal of biofilms in VLU. The evidence for wound washing is heterogeneous, and there is insufficient evidence to suggest that one approach is superior to the other. The NICE medical technology consultation document found that Prontosan® showed promise in chronic ulceration, but recommended that RCTs be performed to assess the clinical and cost effectiveness of this wound washing approach ^23^.

**Endovenous ablation:** This is the standard of care for ulcers for < 6 months, as recommended by national and international guidelines based on the results of the EVRA trial. In EVRA, ulcers > 6 months were excluded, resulting in 1772 (27%) of the screened population being excluded from the trial ^9^. There are no data on the clinical and cost-effectiveness of endovenous ablation for ulcers for > 6 months.

**Duplex ultrasound surveillance:** Duplex ultrasound is the gold standard assessment tool for patients with venous diseases. It provides details on the anatomy and function of the veins in the lower limb. After the intervention, the majority of VLU patients were discharged to their GPs and did not receive follow-up. It is unknown whether intervening in the refluxing veins can prevent ulcer recurrence. The ESCHAR trial found that surgical removal of refluxing veins reduced VLU recurrence rates at 12 months ^10, 11^. Performing duplex ultrasound surveillance and treating any refluxing veins prophylactically may prevent VLU recurrence.

Participants would be randomized to an intervention/comparator according to their domain (clinical presentation, stage C6, or C5).

Domain 1: medication only (pentoxifylline) in addition to SoC; antimicrobial wash only (prontosan) + SoC; combination (pentoxifylline and prontosan) + SoC. Patients will be eligible for superficial endovenous ablation as part of the SoC, and this will not be withheld.

Domain 2: medication only (pentoxifylline) + SoC; superficial endovenous ablation only + SoC; combination (pentoxifylline and superficial endovenous ablation) + SoC.

Domain 3: medication only (pentoxifylline) + SoC; duplex ultrasound surveillance only + SoC; combination (pentoxifylline and surveillance) + SoC.

**Identification of additional platform trial arms:** In WS3 and WS4, we not only identified the technologies to assess in the first version of the platform trial but also potential technologies to assess in future arms. Statin therapy, deep venous intervention, and skin substitutes were identified as the next tier of interventions to be assessed in the proposed platform trial.

**Figure 3.**
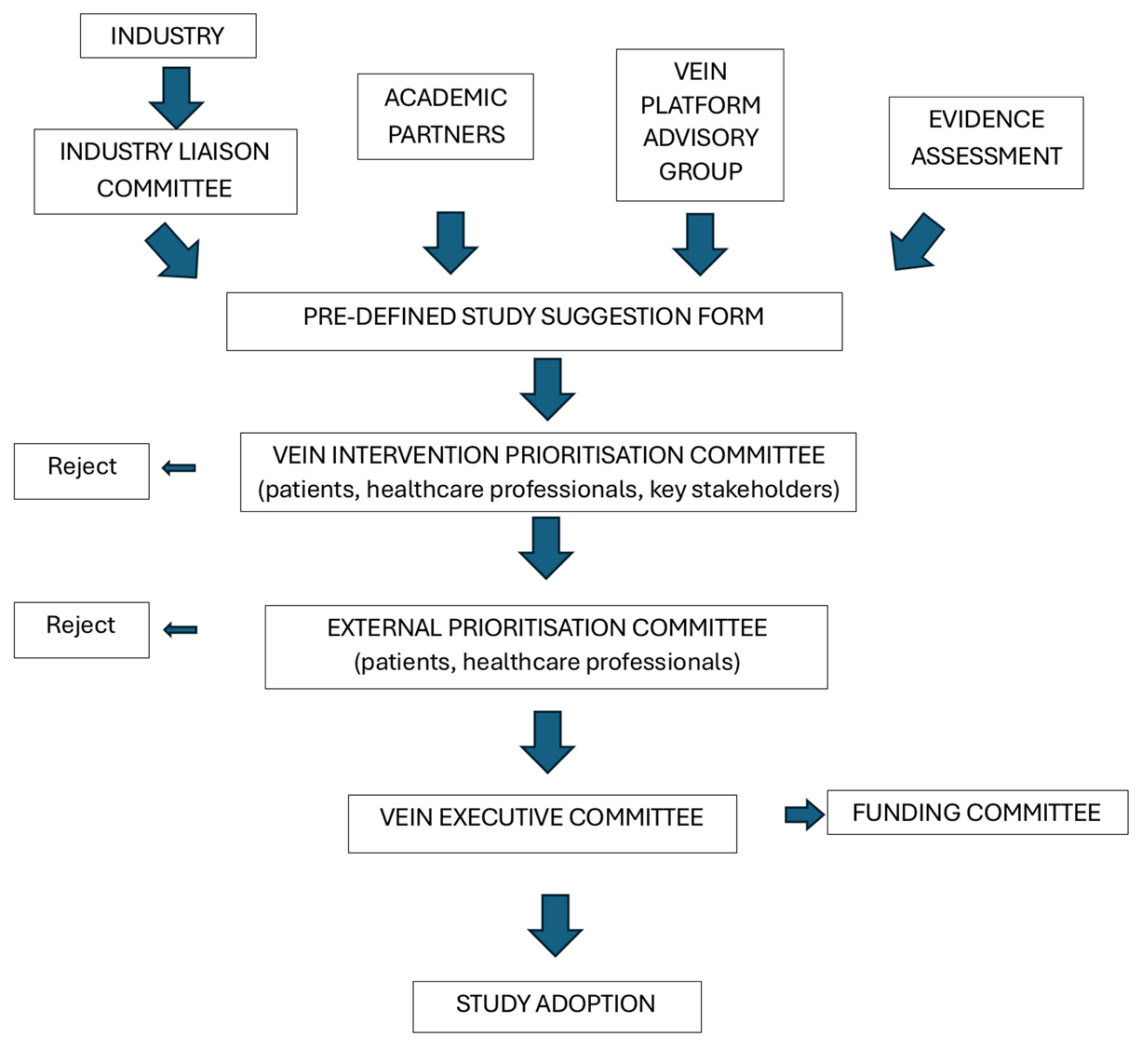
Process for future adoption of VEIN interventions.

In addition, we developed a process for identifying and selecting additional trial arms. We aimed to include a periodical (six monthly) assessment of the literature to identify the latest technologies in venous ulcer care via regular systematic reviews. The results of the reviews would be presented to the VEIN prioritization committee and undergo the process described in Figure 3 to permit the prioritization of the proposed additional arms.

**Sample size, recruitment**: For all three domains, sample sizes were calculated at 90% power and (one-sided) 2.5% significance rate, with a correction for multiple testing applied to account for each therapy being assessed twice within each domain (once individually and once as part of a combination therapy). The resulting family wise error rate (FWER) for each arm was set at 1.25%.

For Domain 1 we estimated an 86% 6-month healing rate with early ablation and 70% without ablation alongside a 10% absolute rate of improvement at 6 months based on the NICE estimate of wounds healing 1.46 – 1.66 times faster with antimicrobial washes. To investigate a 10% increase in healing rate with a 4-arm, 4-stage MAMS, adjusting for a 10% withdrawal rate and inflation to FWER via interim efficacy testing, a maximum of 2244 patients were estimated.

For Domain 2, we anticipated a 40% healing rate in the SoC arm after 6 months, with an estimated 15% absolute treatment effect, as supported by published data ^24^. Based on a SoC 6-month healing rate of 40%, to investigate a 15% increase within a 4 arm, 4 stage MAMS adjusting for a 10% withdrawal rate and inflation to FWER via interim efficacy testing, a maximum of 1320 patients would be required.

For Domain 3, we estimated recurrence rates of 12% in healthy VLU patients with previous interventions. The recurrence rate with compression alone was approximately 30%. The National Wound Care Strategy Programme (NWCSP) anticipated a 19% absolute recurrence rate based on an estimated 58% of patients with a history of VLU undergoing intervention ^12^. Using a time-to-event approach and a SoC 1-year recurrence rate of 19%, a 4 arm, 3 stage MAMS trial to detect a hazard ratio of 0.75 (equivalent to a 4.6% absolute reduction in 1-year recurrence) with a 10% withdrawal rate, would require a maximum of 3395 patients.

Sample sizes were initially derived using the NSTAGE and ARTPEP packages in STATA v17.0 and were supplemented by additional simulation work to optimize both the number and timing of interim analyses.

**Measurement of costs and outcomes:** We planned to develop a platform-wide health cost-effectiveness core model, adapted and reused as required ^25^ for various analyses based on NICE Reference Case specifications ^26^ with flexibility built into the models to allow alternative perspectives and analyses using modular cost and health outcome sets for each CEAP stage. Each domain would contribute data to the wider model, informing the care pathway section they relate to as they report, grow, and refine model outputs over time with unit costs from standard UK sources. A user manual and version control was planned to support staff succession and the creation of a legacy asset. We planned to report model results (QALY based on EQ-5D-5L and costs) alongside within-trial analyses for each domain when it demonstrated superiority or concluded the follow-up. The initial model structure was derived through consultations with clinical experts, an initial broad literature review, and ad hoc focused (non-systematic) reviews to populate specific parameters. Uncertainty was assessed using sensitivity, scenario, and probabilistic sensitivity analyses. To optimize data synthesis and generalizability, a common resource use survey was used in all trials, complemented by Hospital Episode Statistics (HES) and mortality records.

**Time / Follow up:** Telephone consultations from the core lab were planned at the following time points: Domain 1:6, 12, 18, and 24 weeks. Domain 2: 6, 12, 24 weeks. Domain 3:12, 24, and 52 weeks with further follow-up at 12 weekly intervals up to a maximum of 48 months. Long-term follow-up would be performed via data linkage with HES, ONS, and primary care databases (e.g., CPRD). At follow-up: medication check, eCRF completion, EQ5D completion, review and reporting of AEs, SAEs, SUSARs, drug accountability, and compliance. Throughout the trial, ad hoc ulcer healing and recurrence assessment (photographic evidence from patients remotely or face-to-face assessment where required), implementation work, and PPI (facilitated by the implementation team, PPI leads, and the Equality and Diversity lead) were conducted.

**Statistical analysis:** This study was planned based on the intention-to-treat principle. For domains 1 and 2, the primary outcome (proportion of patients with index ulcer healing within 6 months of randomization) was assessed using a logistic regression model. For Domain 3, the primary outcome (time to ipsilateral limb ulcer recurrence from randomization) was assessed using a Cox regression (proportional hazards) model.

Secondary (efficacy) outcomes Quality of life (EQ-5D-5L) data were analyzed by comparing SoC against each trial arm individually using ANCOVA adjusted for baseline, stratification variables, and key clinical covariates. Cumulative data on harm were presented to the data monitoring committee at regular intervals.

A detailed statistical analysis plan was planned.

## Discussion

This is the first international platform trial development exercise in venous leg ulcer research. Through this research, we identified key priorities for research, key stakeholders in developing the research, a finalized project, and a draft proposal for funding. The final proposal was submitted to NIHR for funding. This successfully passed Stage 1 but was rejected for funding in Stage 2 with the following comments:

1. Concerns regarding whether adaptive platform design is required. In addition, the committee was unclear as to how future interventions would be included, and how industry would be involved.
2. Concerns regarding value for money.
3. Concerns regarding the primary outcome and how it would be assessed.
4. Concerns regarding deliver ability and whether enough resources had been allocated to primary care.
5. Concerns regarding fidelity for some interventions.

Despite this, this study has highlighted several important points for VLU research.

- Key priorities for VLU research
- Clear sample sizes/ plans to deliver complex trials in VLU
- Treatments / interventions with poor evidence base that require urgent assessment

We will look for further funding opportunities for this platform trial and work on the feedback received by the NIHR prior to resubmission. Specifically, we will clarify the VEIN prioritization process for novel arms, define the primary outcome in greater detail, and provide reassurance regarding intervention fidelity. We will review the costs and provide modelling for projected cost effectiveness to justify the value for money and resource allocation.

## Ethics and consent

This was a grant development and service evaluation exercise, exercise, so no ethical approval to consent in the research was required.

## Data Availability

No data are associated with this article.
