# Supplementary material for: Designing a multi-arm, multi-stage platform trial for venous leg ulceration – Venous leg ulcers: management and eradication, the VEIN platform study. Designing a platform/adaptive trial for venous leg ulcers (VLU) – The VEIN platform
Source: NIHR Open Res. 2025 Apr 14;5:32. [Version 1] doi: 10.3310/nihropenres.13847.1 (PMC12141998; doi:10.3310/nihropenres.13847.1)
Supplement: Peer review history

# Reviewer report: Reviewer response for version 1

**DOI:** 10.3310/nihropenres.15048.r35391

**Published:** 2025-06-13

**Authors:** E. Andrea Nelson

**Affiliations:** 1 Glasgow Caledonian University, Glasgow, Scotland, UK

ABSTRACT

 For the reader coming to the abstract, it is not immediately obvious that this paper describes the researchers’ journey towards a trial platform which was not funded.   In the Abstract it would help if the tense was consistent – for example in results it states, ‘trial will be delivered’, whereas the trial was planned to be delivered is more accurate.

 It was great to see a set of authors describe the pathway to their trial development. The abstract conclusion states that many opportunities in methodology and challenges were identified – and I think that the paper would be strengthened by specifically setting out those opportunities and challenges. The discussion is brief and could be expanded to cover these.

 PLAIN LANGUAGE SUMMARY (PLS)

 The plain language summary is well written.  As per the observation above – it would be helpful to keep the tense of the PLS consistent – as it stated that ‘...we tested these different treatments.  The tense should be the future historic – we planned to test these different treatments.

 The question posed in the PLS does not look right to me.  In italics it is phrased as ‘ *what is the ideal study to test multiple different treatments for venous leg ulcers? * From my reading the main question that the authors are posing isn’t ‘what is the ideal study design’ – as they do not look at various study designs for comparisons sake – rather the question is about identifying approaches to VLU treatment and prevention, and can they be combined in an efficient manner to answer several questions at once, replicating the complexity of care experienced by these patients, and providing good quality trial design.

 INTRODUCTION

 The role of compression appears to be underplayed (versus surgery) in paragraph 2 – and a reader might expect a comment on the type of compression therapy that might form the standard of care (SOC) in the various arms of the study.

 In the introduction thought that a brief description of a platform trial would have been helpful.   For example, how it differed from not only a standard parallel trial, but a factorial trial.  Indeed, there has been a factorial 2 by 2 by 2 trials of VLU treatment undertaken previously which sought to consider wound healing by randomising to 1 of 2 dressings, 1 of 2 compression systems, and pentoxifylline or placebo.  It would be interesting in the introduction to set out the added benefit from platform trials over such previously used approaches, for example.

 METHODS

 It would be helpful to understand who was involved in PPI as the text says ‘all patients were involved in the development of this study’ and I suspect that the authors convened a group of patients who contributed (all of them) rather than inferring that all VLU patients contributed – as they clearly didn’t.

 Workstream 1 – is there any planned output form your review of the existing literature? That would seem to be a very helpful output from the work involved.

 Workstream 2 – regards the SPAG for industry – what were the protections put in place to ensure that there was a consideration of evidence from industry which was reviewed for validity and reliability regardless of source (large company / SME etc). 

 Re suggestions for industry-developed interventions (as opposed to interventions such as care delivery approaches)  - how was it determined whether these were AMAS ready?  For example, was there a de minimus position for evidence of safety and effectiveness before they could be considered for the MAMS?

 Workstream 3 – (page 6 of pdf) sentence one appears tautologous.   It is not clear what the goal of the focus groups and interviews was if it wasn’t to collect information on priorities - and as described in the manuscript it would appear that they were used to ‘inform them’.  Is this simply a typo error (as removing the word ‘them’ would help ensure that the data collection was from PPI participants to the trial team and not the reverse.

 Workstream 4 – in one place (page 5) there is a typo in the header “consensus on the optimal design’ – and not ‘consensus one’

 There is a missing T = Timeline in the PICOT bullet point on page 5 of the pdf in WS4.

 RESULTS

 What is a complex RCT? See above comment regards factorial trial in this area.

 It would be helpful to understand what threshold the investigators considered had to be met for an intervention to be considered for a MAMS.   I would suspect that there needed to be a level of evidence of effectiveness, but not sufficient so that the intervention was already considered ‘standard / widely recommended’.  Furthermore, an intervention would likely only be recommended for the MAMS if there was a reasonable amount if evidence to establish potential effectiveness in some high quality studies.  

 For pentoxifylline, for example, what was the reasoning behind saying that more evidence was needed (given the systematic review evidence base – Jull et al)?

 For the cleansing approach described (Prontosan) – what was the eligibility framework that determined that this approach would be the most appropriate (in terms of value of information) to be chosen?  There are a number of approaches to wound cleansing which have been proposed over the years for VLU and hence Rx such as water jet therapies for example, might also have partial evidence of benefit.  For the MAMS to be understood in terms of what comes next, it would be important to flesh out what the approach was to intervention selection with more detail.  This is particularly as Industry is involved.   What if the top ‘value of information’ candidate interventions were NOT new medical devices (for example exercise / leg ulcer clinics / surveillance), then what approach would be taken?   Is the evidence lack for this only in the community – in which case what is the reason for testing across all settings?

 In terms of the interventions regards surgery the EVRA trials tested this is people with ulceration for less than 6 months – and hence it is determined that testing in people > 6 months would be useful. From the perspective of a logic model is this the most pressing question for that group, given there is nothing to differentiate from ulcers that are 5.5 months old and 6.5 months old (and the reporting of these boundaries likely variable), so it is not clear why 6 + months ulceration is deemed a different subset than <6 months (would it not make sense to stratify by duration / build into regression model?)

 It was not clear (page 8/13 in the pdf) what other long terms morbidity outcomes would be harvested (when and what).

 I may have missed what the standard of care was for the prevention and the treatment arms – it deserves making very clear what they are. For example the level of compression may impact recurrence (Cochrane review). 

 How would the trial have managed to build in a pentoxifylline arm given it is not licenced for that use?   It may be apparent to the authors how it would be managed (? Via a CTIMP) but useful to flag up that this might be one of the aspects of the trial design you had to manage.

 Outcomes – on p9 it is noted that % healed at 6 months is the primary outcomes, and it seems a waste of data to have a dichotomous outcome here rather than a continuous one of time to healing (and hence increased power). Could you say what was the reasoning behind that? 

 Regards healing outcomes – could you say something about why 6 months healed time was chosen?  Is it related to the fact that in a MAMS you need to have an outcome that you can see in a shortish period of time as to be able to collect enough evidence of effect (or none)?  In which case the higher power of continuous data from regular assessment of healed status or not (and time to event analyses) would be possibly helpful. 

 On Page 10 the listing in the text of the 4 arms does not mention the SOC arm anywhere – and it would be useful to do so for completeness, and describe whether SOC is trial ‘set’ or decided at each trial setting? 

 Page 11 – Domain 1:  I did not follow the sample size calculations given the ablation and non-ablation data and the 1.46 to 1.66 healing rate RR for Prontosan, hence my request for statistician review. 

 Domain 2 – not clear what reference 24 refers to here. 

 Domain 3 – the trail size is large and I wonder where they might relate to the largest previous VLU trials (Treatment and prevention) and how long they took across multiple centres. 

 P11 – Figure 3 – relates to point above regards how there was explicit consideration of any potential conflicts of interest when the key stakeholders group might include industry.

 Overall – what are the potential COI for the investigators and authors? Is that elsewhere?

 Page 12 – the ease of obtaining photographic evidence of ulcer status from patients would be an interesting point to understand more about.  Did they pilot this and what were the findings?

 Page 12 - What outcomes were planned from HES / ONS CPRD etc. and over what period ?

Is the work clearly and accurately presented and does it cite the current literature?

Partly

If applicable, is the statistical analysis and its interpretation appropriate?

I cannot comment. A qualified statistician is required.

Are all the source data underlying the results available to ensure full reproducibility?

Partly

Is the study design appropriate and is the work technically sound?

Yes

Are the conclusions drawn adequately supported by the results?

Partly

Are sufficient details of methods and analysis provided to allow replication by others?

Partly

Reviewer Expertise:

I am an academic nurse researcher whose work has focussed on venous ulcers, pressure ulcers and diabetic foot ulcers. My methodological approaches have mainly included systematic reviews of the effects of interventions and randomised controlled trials of interventions to prevent or treat chronic wounds and their complications.

I confirm that I have read this submission and believe that I have an appropriate level of expertise to confirm that it is of an acceptable scientific standard, however I have significant reservations, as outlined above.

# Reviewer report: Reviewer response for version 1

**DOI:** 10.3310/nihropenres.15048.r35662

**Published:** 2025-06-05

**Authors:** Sergiu-Ciprian Matei

**Affiliations:** 1 “Victor Babeș” University of Medicine and Pharmacy, Timișoara, Romania

Venous leg ulcers still represent one of the most difficult challenges for phlebologists. Especially in my area, they are frequently encountered, and I deeply understand the importance of a well conducted therapeutic management for these patients/conditions.

 I have read the contents of this paper with interest. The authors proposed a multi-arm multi-stage  platform, highlighting many challenges and opportunities in methodology development and the potential delivery of such a study, helping inform future research in this field.

 I would like to extend to the authors my appreciation for this outstanding work. Congratulations!

 While this is a well written paper which totally accomplishes the scientific rigors and provides novelty and interests for the readers, I do not have additional comments. My only recommendation is related to a strategy in order to spread such platform, and to include more experts/researchers from different areas.

Is the work clearly and accurately presented and does it cite the current literature?

Yes

If applicable, is the statistical analysis and its interpretation appropriate?

Yes

Are all the source data underlying the results available to ensure full reproducibility?

Yes

Is the study design appropriate and is the work technically sound?

Yes

Are the conclusions drawn adequately supported by the results?

Yes

Are sufficient details of methods and analysis provided to allow replication by others?

Yes

Reviewer Expertise:

Phlebology, general surgery, wound management

I confirm that I have read this submission and believe that I have an appropriate level of expertise to confirm that it is of an acceptable scientific standard.

# Reviewer report: Reviewer response for version 1

**DOI:** 10.3310/nihropenres.15048.r35533

**Published:** 2025-06-03

**Authors:** Yung-Wei Chi

**Affiliations:** 1 University of California, Davis, California, USA

**I applaud the efforts from Drs. Onida, Davies and the VEIN Platform consortium working group for such a detailed outline platform study proposal on VLU.**

 The manuscript has been thoroughly vested due to numbers of experts involved and my only question is the use or adoption of AI (ChatGPT, Deepseek etc) in the design or as a supplement to the overall draft.

Is the work clearly and accurately presented and does it cite the current literature?

Yes

If applicable, is the statistical analysis and its interpretation appropriate?

Yes

Are all the source data underlying the results available to ensure full reproducibility?

Yes

Is the study design appropriate and is the work technically sound?

Yes

Are the conclusions drawn adequately supported by the results?

Yes

Are sufficient details of methods and analysis provided to allow replication by others?

Yes

Reviewer Expertise:

Venous, arterial and lymphatic disease pathologies, diagnosis and management

I confirm that I have read this submission and believe that I have an appropriate level of expertise to confirm that it is of an acceptable scientific standard.
